# The Use of Synthetic Electronic Health Record Data and Deep Learning to Improve Timing of High-Risk Heart Failure Surgical Intervention by Predicting Proximity to Catastrophic Decompensation

**DOI:** 10.3389/fdgth.2020.576945

**Published:** 2020-12-07

**Authors:** Aixia Guo, Randi E. Foraker, Robert M. MacGregor, Faraz M. Masood, Brian P. Cupps, Michael K. Pasque

**Affiliations:** ^1^Institute for Informatics (I^2^), Washington University School of Medicine, St. Louis, MO, United States; ^2^Department of Internal Medicine, Washington University School of Medicine, St. Louis, MO, United States; ^3^Department of Surgery, Washington University School of Medicine, St. Louis, MO, United States

**Keywords:** electronic health record (EHR), machine/deep learning, heart failure, synthetic data, surgical intervention

## Abstract

**Objective:** Although many clinical metrics are associated with proximity to decompensation in heart failure (HF), none are individually accurate enough to risk-stratify HF patients on a patient-by-patient basis. The dire consequences of this inaccuracy in risk stratification have profoundly lowered the clinical threshold for application of high-risk surgical intervention, such as ventricular assist device placement. Machine learning can detect non-intuitive classifier patterns that allow for innovative combination of patient feature predictive capability. A machine learning-based clinical tool to identify proximity to catastrophic HF deterioration on a patient-specific basis would enable more efficient direction of high-risk surgical intervention to those patients who have the most to gain from it, while sparing others. *Synthetic* electronic health record (EHR) data are statistically indistinguishable from the original protected health information, and can be analyzed as if they were original data but without any privacy concerns. We demonstrate that *synthetic* EHR data can be easily accessed and analyzed and are amenable to machine learning analyses.

**Methods:** We developed *synthetic* data from EHR data of 26,575 HF patients admitted to a single institution during the decade ending on 12/31/2018. Twenty-seven clinically-relevant features were synthesized and utilized in supervised deep learning and machine learning algorithms (i.e., deep neural networks [DNN], random forest [RF], and logistic regression [LR]) to explore their ability to predict 1-year mortality by five-fold cross validation methods. We conducted analyses leveraging features from prior to/at and after/at the time of HF diagnosis.

**Results:** The area under the receiver operating curve (AUC) was used to evaluate the performance of the three models: the mean AUC was 0.80 for DNN, 0.72 for RF, and 0.74 for LR. Age, creatinine, body mass index, and blood pressure levels were especially important features in predicting death within 1-year among HF patients.

**Conclusions:** Machine learning models have considerable potential to improve accuracy in mortality prediction, such that high-risk surgical intervention can be applied only in those patients who stand to benefit from it. Access to EHR-based synthetic data derivatives eliminates risk of exposure of EHR data, speeds time-to-insight, and facilitates data sharing. As more clinical, imaging, and contractile features with proven predictive capability are added to these models, the development of a clinical tool to assist in timing of intervention in surgical candidates may be possible.

## Introduction

Heart failure (HF) patients comprise the largest, most rapidly growing, and most expensive subset of patients with cardiovascular disease^1^. In the early stages of new-onset HF, the clinical prediction of each patient's potential for a favorable response to medical therapy is critical since it determines initial management and sets the stage for their ultimate clinical course. This prediction is confounded by the fact that these patients commonly present in profound clinical HF with severely impaired left ventricular (LV) function (ejection fraction <20%) (1), only to subsequently demonstrate a very favorable response to medical therapy. Despite the gravity of their initial presentation, they are essentially cured by medical therapy alone. Conversely, many patients with an identical clinical presentation do in fact suffer precipitous deterioration (2).

Unfortunately, the poor prognostic performance of the qualitative metrics (echocardiographic, functional, metabolic, and others) that currently drive HF therapeutic clinical algorithms leaves little hope of accurate one-on-one individual patient risk-stratification (3). In fact, because of the lack of metrics that can accurately and reliably predict catastrophic hemodynamic deterioration, many HF programs have adopted a very low threshold for early and highly invasive surgical intervention (4). Thus, upon initial presentation with profound LV impairment, congestive symptoms, and borderline hemodynamics, new-onset HF patients are often rushed off to invasive surgery for intra-aortic balloon pump, extracorporeal membrane oxygenator (ECMO) support, or ventricular assist device (VAD) placement with immediate listing for cardiac transplantation (2). It is tragic to subject patients to the significant risks of surgical intervention if they can be managed on medical therapy alone. Similarly, however, over-compensating toward medical therapy in these critically ill patients also has a major downside: we are equally unable to determine which of these patients will suddenly deteriorate while on medical therapy. This deterioration is often so rapid and unheralded that sudden death or severe end-organ failure preclude any further efforts (5). All too often, we are left with patients whose “windows of opportunity” have passed under our watch.

Thus, our inability to accurately and consistently differentiate these two patient subsets at the time of presentation results in high-risk surgery being unnecessarily applied to some patients, while being denied to others who have the most to gain from it. Improving the accuracy of the metrics utilized to predict response to guideline-directed medical therapy has obvious potential to more accurately direct the clinical use of highly invasive, risky, and expensive HF surgical intervention. We seek to more accurately identify HF medical therapy *non-responders* on a one-by-one basis. This would enable their targeting for intense surveillance with an appropriately lowered threshold for early evaluation for high-risk therapy—while simultaneously sparing those who will ultimately respond to lower-risk medical therapy.

Machine learning can detect non-intuitive classifier patterns that allow for innovative combination of patient feature predictive capability (6). Recently, deep learning algorithms have been successfully used in electronic health record (EHR) data from healthcare fields. Deep learning algorithms can effectively capture the informative and useful features and patterns from the rich healthcare information in EHR data (7). For example, a very recent study showed that deep-learning-based model achieved significantly higher accuracy to predict mortality among acute heart failure patients than the existing score models and several machine learning models by using EHR data (8–13).

One of the problems with deep learning applications in heart failure is the management of large volumes of incomplete EHR information. The specter of public exposure of protected individual patient health information is also an important consideration when accessing the often-massive datasets commonly used in deep learning analysis of healthcare information (14). In regard to these concerns, *synthetic* electronic health record (EHR) data are statistically indistinguishable from that of original protected health information, and can be analyzed as if they were original data but without any privacy concerns (15).

In this investigation, we utilize an entirely synthetic dataset derived from a large cohort of HF patients seen at a single institution to test several machine learning methodologies regarding their prediction of HF outcomes. Using entirely synthetic data, we developed and compared a deep learning model—deep neural networks (DNN) (16)—with two machine learning models—random forest (RF) (17) and logistic regression (LR) (18)—to predict 1 year mortality among heart failure patients. Feature importance determinations by a tree-based classifier (19) were utilized to optimize comparison of model performance.

## Methods

### Data Source and Study Design

In this study, the electronic health records (EHR) data was from a single hospital, Barnes-Jewish Hospital from a large academic medical center, Washington University in St Louis. These data were synthesized by MDClone platform, which can create synthetic electronic health data that is statistically equivalent to original data, but contains no actual patient information^2^. The synthetic data generation platform creates a computationally derived data set which is statistically identical to that of the original patients. The computationally-derived variables and their pairwise correlations had the same or very similar distributions as the relationships among variables in the original data (20). We included a Spearman's correlation comparison between the variables in the original compared to the variables derived from the MDClone synthetic data platform (Supplementary Figure 1). The original patient cohort, from which the synthetic data was derived, were admitted for treatment at Barnes-Jewish Hospital with an admitting diagnosis of heart failure during the decade ending on 12/31/2018. Our goal was to predict their proximity to catastrophic HF decompensation by predicting 1-year mortality based upon features contained in their EHR after/at or prior to/at the earliest diagnoses of heart failure. We studied 26,575 (26,600) patients if using features prior to/at (if after/at) heart failure diagnoses.

For the feature extraction, we discarded features whose missing values rate exceeded 70%, as we expected that they may cause a substantial difference between features available prior to/at and after/at the time of HF diagnosis. For example, the feature “CABG—Procedure code” was included in the case of after/at HF diagnosis, but was excluded from the case of prior to/at HF diagnosis as it had a missing value rate more than 70%. For all others, we imputed any missing values as the mean value for the continuous variables and the mode value for the categorical variables. Under the criteria, there were 27 features and one outcome (death) were included in our study. The included features and possible value examples for each feature were listed in Table 1.

**Table 1:** Included 27 features and examples of feature values.

**Feature names**	**Feature description and value examples**
Gender	Gender (e.g., Female, Male)
Primary race	Race (e.g., White, Black, Asian, Other)
Age at event	Age of patients when the first time diagnosed with HF
Visit group	Visit types (e.g., Inpatient visit, Outpatient visit, Emergency room visit, Observation Same day Visit, Ancillary, Pre-visit, Series)
Source diagnosis	Diagnosis types (e.g., Cardiomyopathy, unspecified, Dilated cardiomyopathy, Other cardiomyopathies, Secondary cardiomyopathy, unspecified', Cardiomyopathy due to drug and external agent', Cardiomyopathy in other diseases classified elsewhere, Alcoholic cardiomyopathy, Cardiomyopathy in diseases classified elsewhere Nutritional and metabolic cardiomyopathy)
Diagnosis type	Diagnosis types (e.g., Final Diagnosis, Admitting, Reason for visit, Interim)
Facility	Facility (e.g., BJC/Washington University)
Present on admission	If HF present on admission (e.g., Yes, No, Ns)
Principal problem	If HF is the principal problem (e.g., True, False)
Problem class	Problem class (e.g., Chronic, Temporary)
Severity	Severity (e.g., High)
BMI-Age at measurement	Age of patients at the measure of BMI
BMI-Average calculated bmi	The numeric value of BMI
BP-Age at Measurement	Age of patients at the measure of BP
BP-Diastolic	The numeric value of BP Diastolic
BP-Systolic	The numeric value of BP Systolic
Steroids-Age at medication order	Age of patients at the order date of Steroids
VHD-Condition	Valvular heart disease (VHD) (e.g., Endocarditis, valve unspecified, unspecified cause, Endocarditis, valve unspecified)
Echo-Surgery code	Echocardiogram (Echo) (e.g., 1070001163)
kidD-Age at event	Kidney disease (KidD) (e.g., Chronic kidney disease, stage 3 (moderate), Hypertensive chronic kidney disease, unspecified, with chronic kidney disease stage I through stage IV, or unspecified, Hypertensive heart and chronic kidney disease with heart failure and stage 1 through stage 4 chronic kidney disease, or unspecified chronic kidney disease, Chronic kidney disease, unspecified, End stage renal disease, Hypertensive chronic kidney disease with stage 1 through stage 4 chronic kidney disease, or unspecified chronic kidney disease, Chronic kidney disease, Stage III (moderate), Chronic kidney disease, stage 2 (mild), Cystic kidney disease, unspecified)
creatinine-Age at event	Age of patients at the measure of creatinine
creatinine-Result value numeric	The numeric value of creatinine
SMK-Smoking tobacco status	Smoking (SMK) status (e.g., Former Smoker, Never Assessed, Never Smoker, Current Every Day Smoker, Unknown If Ever Smoked, Heavy Tobacco Smoker, Smoker, Current Status Unknown)
SMK-Age at event	Age of patients at the smoking
AF-Condition	Atrial fibrillation (AF) (e.g., Atrial fibrillation, Paroxysmal atrial fibrillation, Unspecified atrial fibrillation, Chronic atrial fibrillation, Persistent atrial fibrillation)
AF-Age at event	Age of patients at the diagnosis of AF
diab	• Diabetes (diab)—Identify diabetes presented based on if one of the following presented. • Fasting gluecose • Hemoglobin A1c • Diagnosis (e.g., Diabetes mellitus without mention of complication, type II or unspecified type, not stated as uncontrolled, Type 2 diabetes mellitus without complications)

We classified the heart failure patients into two groups based upon their mortality dates: a positive class (patients who died within 365 days of initial HF presentation) and a negative class (patients who did not die or died later than 365 days after HF presentation). There were 1,768 (1,735) positive patients and 24,807 (24,865) negative patients if using features prior to/at (after/at) the first heart failure diagnosis dates.

### Statistical Analysis

We then applied machine learning and deep learning models to predict the all-cause mortality within 1 year by using features either prior to/at or after/at heart failure diagnoses. The three models employed were deep neural networks (DNN), random forest (RF) and logistic regression (LR). For each model of each prediction, we utilized five-fold cross validation by dividing the dataset into five-folds, with each fold serving as a test dataset and the remaining four-folds comprising a training dataset. There was a significant imbalance between the positive and negative classes. We utilized Synthetic Minority Over-sampling Technique (SMOTE) (21) to deal with the imbalanced issue by oversampling positive patients to the same amount of negative patients in each cross validation, i.e., the four-folds training datasets was oversampled by SMOTE while the remaining one-fold which served as testing dataset kept as original without using SMOTE to oversample.

Our DNN was comprised of an input layer (with 27 dimensions), 5 hidden layers (with 256, 256, 128, 64, and 32 dimensions, respectively) and a scalar output layer. We used the Sigmoid function (22) at the output layer and ReLu function (23) at each hidden layer. Binary cross-entropy was used as loss function and Adam optimizer (24) was used to optimize the models with a mini-batch size of 64 samples. The hyperparameter of network depth was searched from 2 to 8 hidden layers. To avoid overfitting, an early stopping technique was used which would stop training when the monitored loss metric stopped improving after 5 epochs. We set the maximum epochs at 50. The LR and RF models were configured by the default options in package of Scikit-learn in Python 3. We performed a grid search of hyperparameters for the RF model by five-fold cross validation. We searched the number of trees in the forest for 100, 200, 500, and 700, and we considered the number of features for the best split according to auto, sqrt, and log2. We also did a grid search of hyperparameter tuning for LR models by five-fold cross validation. In penalization, we searched the norm for L1 and L2 norm, and the inverse value of regularization strength for 10 different numbers spaced evenly on a log scale of [0, 4]. We achieved the best hyperparameters on the default configurations for both RF and LR models.

**Figure 1:**
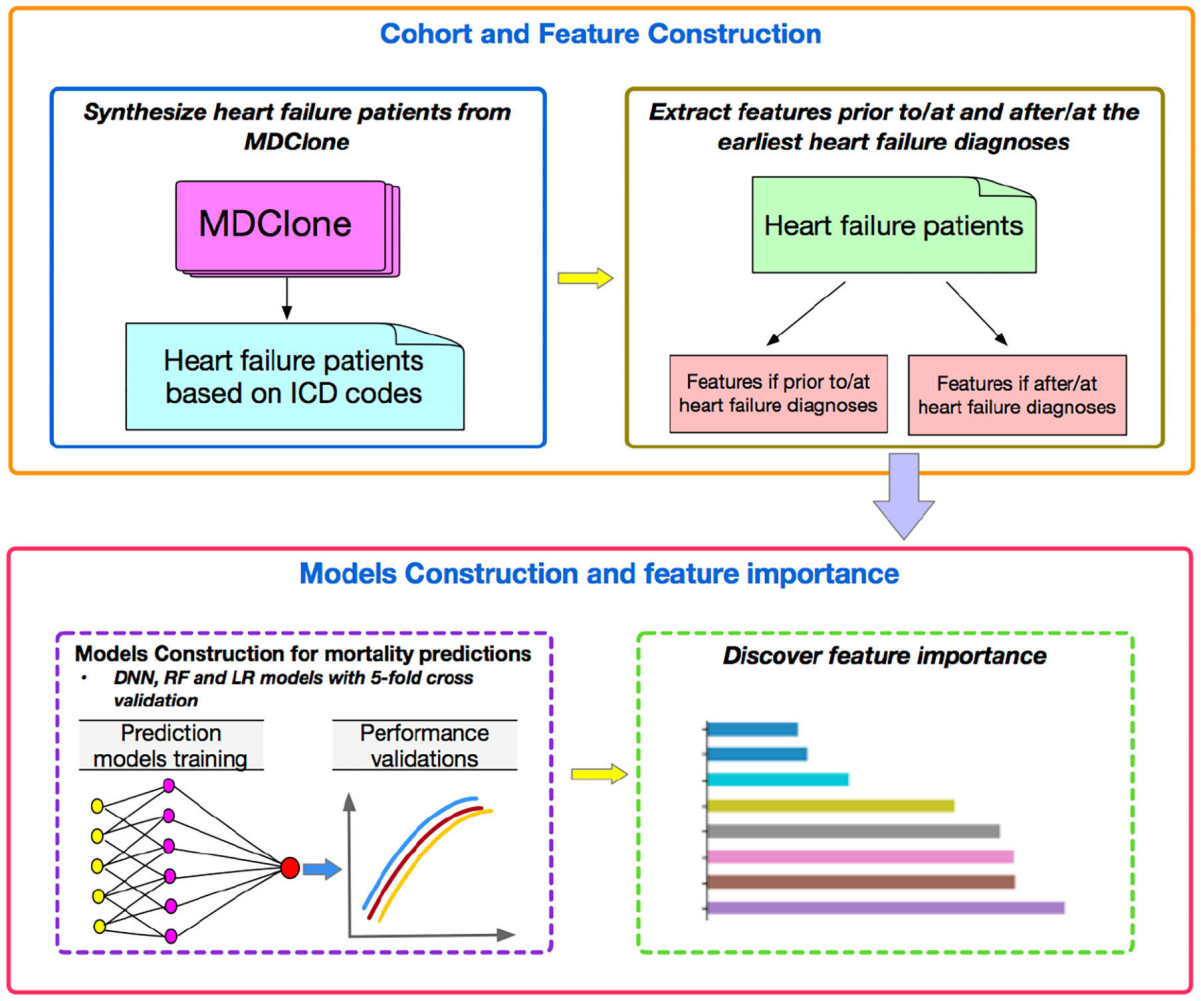
The Flowchart of our work.

Finally, we investigated the feature importance to better understand which features played more important roles compared to others by tree-based classifiers. We quantified the importance of features by ordering them in an ascending order. The prediction performances were then validated by using different numbers of top features in the three machine learning models. Figure 1 represents a flowchart of our data analysis. Analyses were conducted by using the libraries of Scikit-learn, Keras, Scipy, Matplotlib with Python, version 3.6.5 (2019).

## Results

The average age for the two study populations was 63 (Table 2). Approximately 58% of patients in both groups were male and white. There were 327 patients (388 for prior to/at heart failure) who also had a diagnosis of valvular heart disease (VHD) and 14% (19% for prior to/at heart failure) of the patients had diabetes. The average creatinine level was 1.63 (1.41 for prior to/at heart failure) for patients. Approximately 7% of the patients of both study populations died within 1 year from the earliest diagnosis of heart failure.

**Table 2:** Characteristics [mean (SD) or *n* (%)] of the two study populations.

**Demographics**	**After/at heart failure (*n* = 26,600)**	**Prior to/at heart failure (*n* = 26,575)**
Age	63 (17)	63 (17)
Gender		
Female	11,116 (41.8)	11,103 (41.8)
Male	15,484 (58.2)	15,418 (58.0)
Race		
White	15,218 (57.2)	15,420 (58.0)
Black	4,738 (17.8)	5,015 (18.9)
Other/unknown	6,644 (25.0)	6,140 (23.1)
BMI	29.6 (6.3)	29.8 (6.2)
Diastolic blood pressure (DBP, mmHg)	73 (15)	75 (15)
Systolic blood pressure (SBP, mmHg)	127 (23)	131 (23)
Valvular heart disease (VHD) present	327 (1.2)	388 (1.5)
Echocardiogram (ECHO) present	38 (0.1)	5 (0.0)
Creatinine level	1.63 (1.01)	1.41 (0.88)
Current smoker	703 (2.6)	191 (0.7)
Diabetes present	3,809 (14.3)	5,174 (19.5)

**Figure 2:**
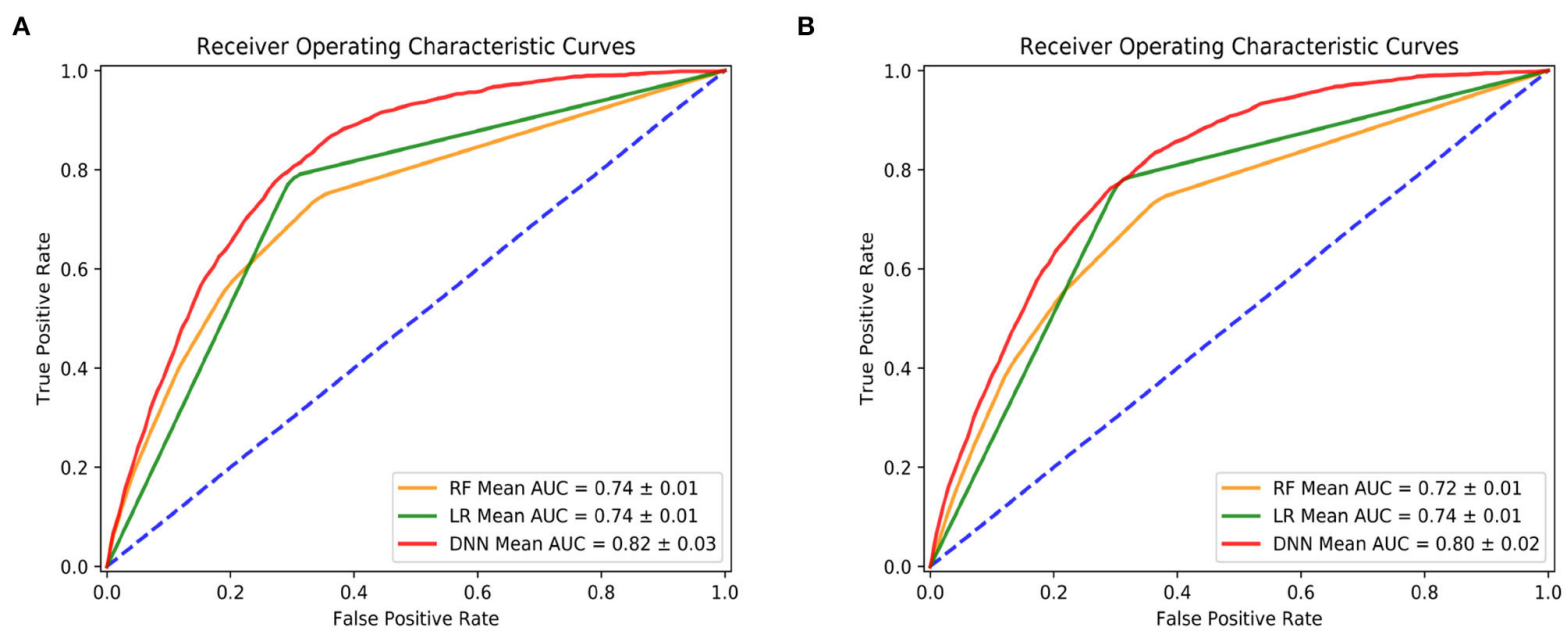
Prediction performance by deep neural network (DNN), random forest (RF) and logistic regression (LR). **(A)** Is using features after and at heart failure diagnoses date; **(B)** is using features prior to and at heart failure diagnoses date.

Figure 2 shows the prediction performance for 1-year mortality by using after/at and prior to/at first diagnosis heart failure start date. All the 27 features are used for these predictions. In the two study groups, DNN models outperformed the other two models of RF and LR and achieved the highest AUC values: the mean AUC value of DNN was 0.82 (0.80) compare to RF and LR with 0.74 (0.72) and 0.74 (0.74) in the five-fold cross validation models.

**Figure 3:**
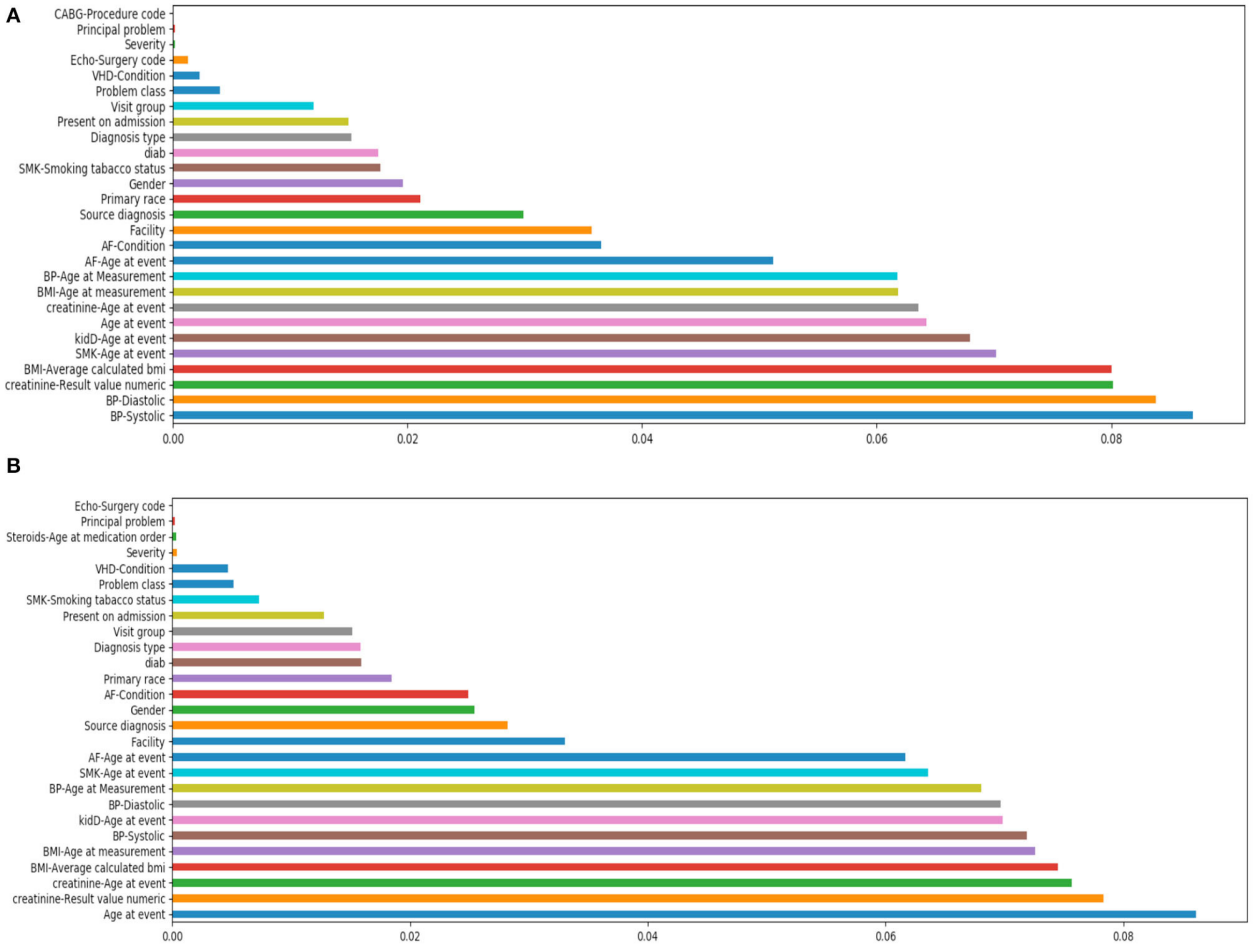
Feature importance study by tree-based classifier. **(A)** Is using features after/at heart failure diagnoses date; **(B)** is using features prior to/at heart failure diagnoses date.

Figure 3 shows the feature importance by the tree-based classifier method for both cases. In the first case of after/at heart failure diagnosis, it shows that the most important features included blood pressure, creatinine levels, body mass index (BMI) etc. In the case of prior to/at heart failure diagnosis, the most important features were age at the first diagnosis of heart failure, creatinine, and BMI.

**Figure 4:**
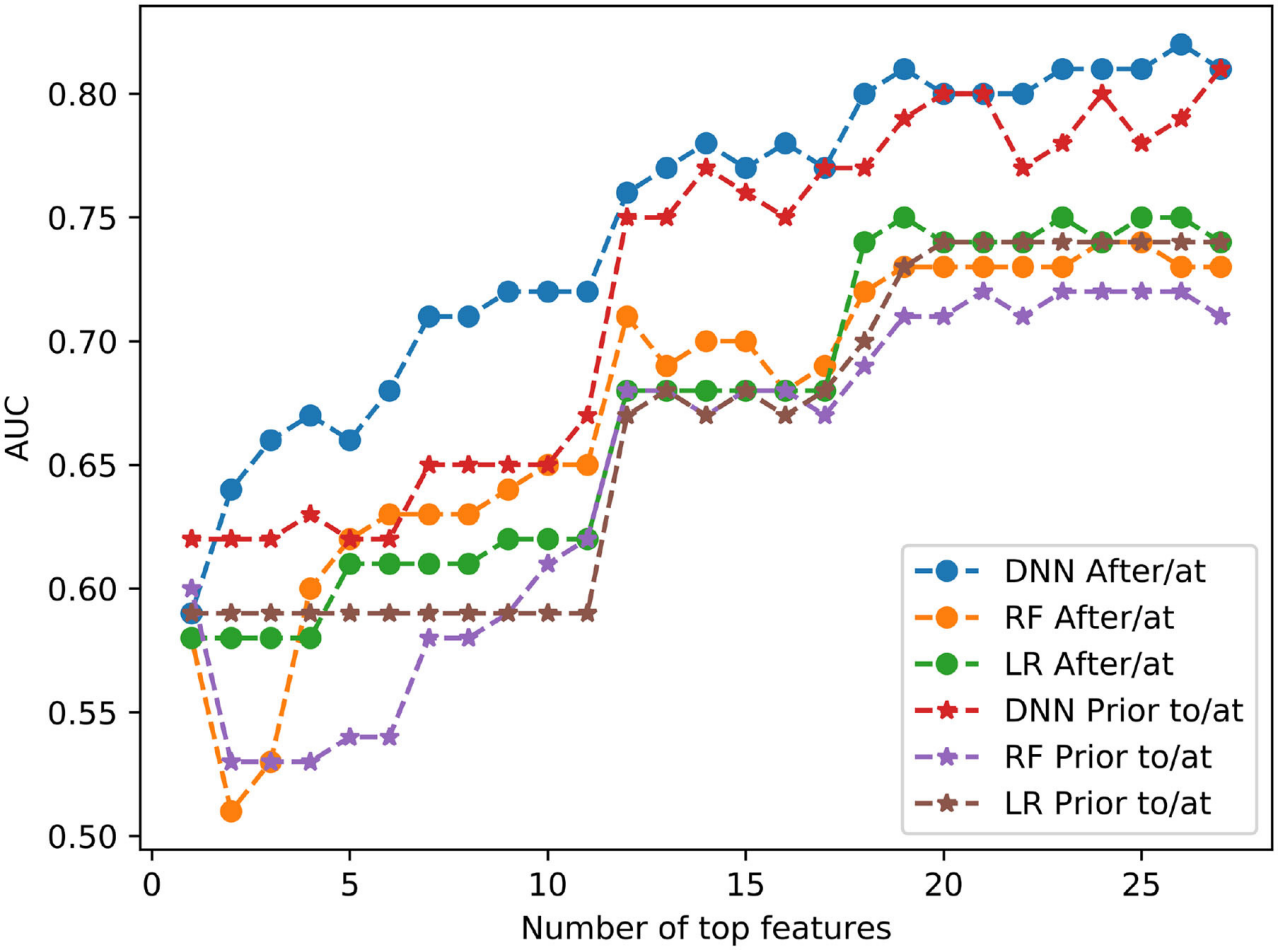
Model performance with different numbers of top features for DNN, RF and LR.

Figure 4 shows the prediction performance by using all different numbers of top features for 3 models of DNN, RF and LR. For example, if # of top feature = 12, it means the models used only the top 12 important features listed in Figure 3 in each case. In all cases, DNN models outperformed RF and LR models. The AUC values were markedly reduced in both study groups when the features dropped from 12 to 11, for all three machine learning models.

## Discussion

In this study, we utilized 10-year synthetic EHR data by MDClone platform to identify heart failure patients to predict the mortality of patients within 1 year from the first diagnoses of heart failure by machine learning and deep learning models. We also investigated the top important features by tree-based classifier and tested all different possible numbers of top features as the inputs for all the three models in both two cases.

Our results indicated that the deep learning model DNN can effectively predict the mortality within 1 year of patients by using features such as measurements and diagnoses from either after/at or prior to/at the first diagnoses of heart failure. Our results also indicated that features such as blood pressure, BMI and creatinine levels are the most informative ones, and in all cases DNN models outperformed RF and LR models. Three models consistently indicated that there was a significant reduction in accuracy of model prediction, as represented by AUC values, when the number of most important features utilized in the model were reduced from 12 to 11, suggesting that 12 features would be a potential threshold if a reduction in features is necessary.

The case of using features from prior to/at HF diagnosis was to provide insights into the 1-year mortality prediction at the time of HF diagnosis, in which a mortality prediction risk score was calculated for patients at the time of HF diagnosis. The case of using features from after/at HF diagnosis to enhance the 1-year mortality prediction following HF diagnosis. At each follow-up time point, a predicted 1-year mortality risk score could be calculated for patients. Based on these scores, providers may make particular treatment decisions to optimize prevention and more effectively manage these patients.

The use of synthetic EHR data in deep learning models to predict 1-year mortality among heart failure patients is unique to this investigation, which also emphasized the use of feature importance to guide mechanistic hypotheses in this HF patient population. This use of synthetic EHR data containing no protected health information uniquely allows a broader application of our results by enabling the sharing of data without risk of exposure of individual patient EHR information. In future work, we plan to pursue additional statistical analyses such as permutation tests and statistical comparisons to investigate the impact of feature importance. We acknowledge that our current DNN model had a relatively simple structure with 5 hidden layers. In future work, we will investigate more complicated structures of DNN models with more hidden layers (e.g., from 2 to 32) and evaluate other novel deep learning models.

## Limitations

This study is limited by the small number of health-related features included in our machine learning applications. Many features were not used in our models because of a high proportion of missing values. As the EHR continues to expand health data inclusion and improve in the accuracy, consistency, and completeness of the data included, model performance will almost assuredly improve by the inclusion of clinical variables with proven predictive capability.

## Conclusions

Machine learning models have obvious and considerable potential to improve accuracy in the risk stratification of HF patients. The ability to use EHR variables to identify HF patient proximity to HF decompensation and death would allow the more accurate and timely application of high-risk surgical intervention. Access to synthetic data derivatives speeds time-to-insight using EHR data, and allows the sharing of massive datasets—while simultaneously reducing privacy concerns by eliminating the risk of personal data exposure. As the EHR becomes more complete, the inclusion of advanced clinical, imaging, and contractile features—with proven predictive capability—in predictive machine learning models can be expected to improve their accuracy. As the accuracy of machine learning, and especially deep learning, models improves, the development of a clinical tool capable of assisting clinicians in the timing of intervention in surgical candidates may be possible. Further, our ability to quantify individual EHR feature impact on mortality prediction may allow the generation of non-intuitive mechanistic hypotheses leading to potential preventative clinical intervention.

## Data Availability Statement

The datasets for the current study are available from the corresponding author on reasonable request. Requests to access these datasets should be directed to aixia.guo@wustl.edu.

## Ethics Statement

Ethical approval was not provided for this study on human participants because synthetic electronic health data that contains no actual patient information was used. The ethics committee waived the requirement of written informed consent for participation.

## Author Contributions

RF contributed to the study design. AG conducted the analysis and wrote the manuscript. RM, FM, BC, and MP provided insightful discussions, reviewed the results and revised the manuscript. All authors contributed to the article and approved the submitted version.

## Conflict of Interest

The authors declare that the research was conducted in the absence of any commercial or financial relationships that could be construed as a potential conflict of interest.

## Footnotes

^1^Available online at: www.americanheart.org

^2^ https://www.mdclone.com/
