# Supplementary material for: The Use of Synthetic Electronic Health Record Data and Deep Learning to Improve Timing of High-Risk Heart Failure Surgical Intervention by Predicting Proximity to Catastrophic Decompensation
Source: Front Digit Health. 2020 Dec 7;2:576945. doi: 10.3389/fdgth.2020.576945 (PMC8521851; doi:10.3389/fdgth.2020.576945)
Supplement: Supplementary file 1 [file Image_1.pdf]

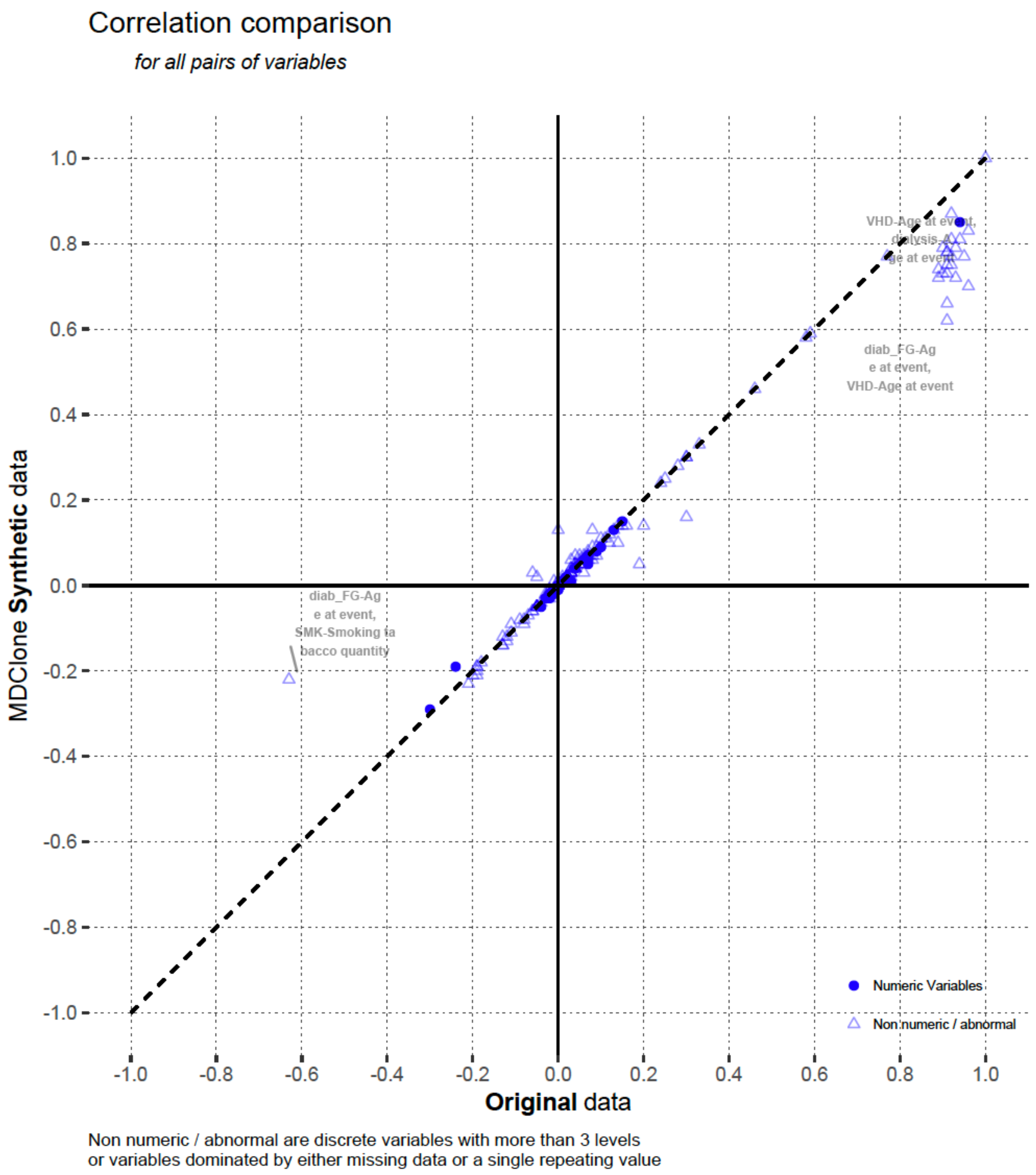

Figure S1. Correlation comparison between variables in the original data and variables in MDClone synthetic data. When the correlations in the synthetic data are identical to the correlations in the original data they are on the diagonal.
