# Supplementary material for: The Use of Synthetic Electronic Health Record Data and Deep Learning to Improve Timing of High-Risk Heart Failure Surgical Intervention by Predicting Proximity to Catastrophic Decompensation
Source: Front Digit Health. 2020 Dec 7;2:576945. doi: 10.3389/fdgth.2020.576945 (PMC8521851; doi:10.3389/fdgth.2020.576945)
Supplement: Supplementary file 2 [file Table_1.docx]

**Table S1. Model performance with different numbers of top features for DNN, RF and LR.**

|  | After/at heart failure features (AUC) | | | Prior to/at heart failure features (AUC) | | |
| --- | --- | --- | --- | --- | --- | --- |
| # of top features | DNN | RF | LR | DNN | RF | LR |
| 27 | 0.82 (0.03) | 0.74 (0.01) | 0.74 (0.01) | 0.80 (0.02) | 0.72 (0.01) | 0.74 (0.01) |
| 26 | 0.82 (0.03) | 0.73 (0.01) | 0.75 (0.01) | 0.79 (0.05) | 0.72 (0.02) | 0.74 (0.01) |
| 25 | 0.81 (0.03) | 0.74 (0.01) | 0.75 (0.01) | 0.78 (0.02) | 0.72 (0.01) | 0.74 (0.01) |
| 24 | 0.81 (0.03) | 0.74 (0.01) | 0.74 (0.02) | 0.8 (0.05) | 0.72 (0.01) | 0.74 (0.0) |
| 23 | 0.81 (0.04) | 0.73 (0.02) | 0.75 (0.01) | 0.78 (0.06) | 0.72 (0.01) | 0.74 (0.01) |
| 22 | 0.8 (0.04) | 0.73 (0.01) | 0.74 (0.01) | 0.77 (0.02) | 0.71 (0.01) | 0.74 (0.01) |
| 21 | 0.8 (0.03) | 0.73 (0.02) | 0.74 (0.02) | 0.8 (0.05) | 0.72 (0.02) | 0.74 (0.01) |
| 20 | 0.8 (0.03) | 0.73 (0.01) | 0.74 (0.02) | 0.8 (0.05) | 0.71 (0.01) | 0.74 (0.01) |
| 19 | 0.81 (0.04) | 0.73 (0.01) | 0.75 (0.01) | 0.79 (0.05) | 0.71 (0.0) | 0.73 (0.01) |
| 18 | 0.8 (0.04) | 0.72 (0.02) | 0.74 (0.01) | 0.77 (0.05) | 0.69 (0.01) | 0.7 (0.01) |
| 17 | 0.77 (0.02) | 0.69 (0.01) | 0.68 (0.02) | 0.77 (0.04) | 0.67 (0.01) | 0.68 (0.01) |
| 16 | 0.78 (0.04) | 0.68 (0.01) | 0.68 (0.0) | 0.75 (0.06) | 0.68 (0.02) | 0.67 (0.01) |
| 15 | 0.77 (0.04) | 0.7 (0.01) | 0.68 (0.01) | 0.76 (0.04) | 0.68 (0.01) | 0.68 (0.01) |
| 14 | 0.78 (0.03) | 0.7 (0.01) | 0.68 (0.01) | 0.77 (0.05) | 0.67 (0.02) | 0.67 (0.01) |
| 13 | 0.77 (0.05) | 0.69 (0.01) | 0.68 (0.01) | 0.75 (0.05) | 0.68 (0.01) | 0.68 (0.01) |
| 12 | 0.76 (0.02) | 0.71 (0.01) | 0.68 (0.01) | 0.75 (0.04) | 0.68 (0.01) | 0.67 (0.01) |
| 11 | 0.72 (0.02) | 0.65 (0.02) | 0.62 (0.01) | 0.67 (0.07) | 0.62 (0.01) | 0.59 (0.02) |
| 10 | 0.72 (0.03) | 0.65 (0.01) | 0.62 (0.01) | 0.65 (0.04) | 0.61 (0.03) | 0.59 (0.01) |
| 9 | 0.72 (0.04) | 0.64 (0.01) | 0.62 (0.01) | 0.65 (0.04) | 0.59 (0.02) | 0.59 (0.02) |
| 8 | 0.71 (0.03) | 0.63 (0.01) | 0.61 (0.01) | 0.65 (0.04) | 0.58 (0.02) | 0.59 (0.02) |
| 7 | 0.71 (0.05) | 0.63 (0.01) | 0.61 (0.01) | 0.65 (0.03) | 0.58 (0.02) | 0.59 (0.01) |
| 6 | 0.68 (0.04) | 0.63 (0.01) | 0.61 (0.01) | 0.62 (0.03) | 0.54 (0.02) | 0.59 (0.02) |
| 5 | 0.66 (0.03) | 0.62 (0.02) | 0.61 (0.02) | 0.62 (0.03) | 0.54 (0.02) | 0.59 (0.01) |
| 4 | 0.67 (0.02) | 0.6 (0.02) | 0.58 (0.01) | 0.63 (0.01) | 0.53 (0.01) | 0.59 (0.01) |
| 3 | 0.66 (0.01) | 0.53 (0.02) | 0.58 (0.0) | 0.62 (0.01) | 0.53 (0.02) | 0.59 (0.01) |
| 2 | 0.64 (0.0) | 0.51 (0.01) | 0.58 (0.01) | 0.62 (0.02) | 0.53 (0.02) | 0.59 (0.02) |
| 1 | 0.59 (0.02) | 0.58 (0.01) | 0.58 (0.01) | 0.62 (0.01) | 0.6 (0.01) | 0.59 (0.01) |
